# Feasibility of a 12-Week, Therapist-Independent, Smartphone-Based Biofeedback Treatment for Episodic Migraine in Adults: Single-Center, Open-Label, 1-Armed Trial

**DOI:** 10.2196/59622

**Published:** 2025-06-09

**Authors:** Amalie Christine Poole, Ingunn Grøntveit Winnberg, Melanie Rae Simpson, Anker Stubberud, Kjersti Grøtta Vetvik, Marte-Helene Bjørk, Lise Rystad Øie, Petter Holmboe, Alexander Olsen, Erling Tronvik, Tore Wergeland

**Affiliations:** 1 Norwegian Centre for Headache Research (NorHead), NTNU Norwegian University of Science and Technology, Trondheim, Norway; 2 Department of Neurology and Neurophysiology, St. Olavs University Hospital, Trondheim, Norway, 47 73 59 20 20; 3 Department of Public Health and Nursing, Faculty of Medicine and Health Sciences, NTNU Norwegian University of Science and Technology, Trondheim, Norway; 4 Department of Neuromedicine and Movement Science, NTNU Norwegian University of Science and Technology, Trondheim, Norway; 5 Department of Neurology, Akershus University Hospital, Lørenskog, Norway; 6 Department of Neurology, Haukeland University Hospital, Bergen, Norway; 7 Department of Clinical Medicine, University of Bergen, Haukeland, Norway; 8 Nordic Brain Tech AS, Oslo, Norway; 9 Department of Psychology, NTNU Norwegian University of Science and Technology, Trondheim, Norway; 10 Department of Physical Medicine and Rehabilitation, St. Olavs Hospital, Trondheim University Hospital, Trondheim, Norway

**Keywords:** mHealth, mobile health, app, biofeedback, therapist-independent, behavioral therapy, headache, migraine, neurology, psychophysiology, complementary, psychology, feasibility, safety, usability, adherence, mobile phone

## Abstract

**Background:**

Biofeedback is an established treatment principle for migraine, but home-based therapy with proven efficacy is not available.

**Objective:**

This study aims to assess the feasibility, usability, and safety of 12 weeks of daily use of a novel medical device (Cerebri; Nordic Brain Tech AS) for therapist-independent multimodal biofeedback preventative treatment in adults with episodic migraine.

**Methods:**

In this open-label, one-armed trial, 20 adult participants with episodic migraine used Cerebri for 12 weeks. The primary outcome was the feasibility of the Cerebri system, measured by the level of adherence to daily biofeedback and electronic headache diary (eDiary) entries. Secondary outcomes were safety, usability, and efficacy.

**Results:**

Initial adherence to biofeedback was high (16/20, 80% in weeks 1‐4), declining to 20% (4/20) by weeks 9‐12. eDiary adherence remained high (15/20, 75% in weeks 9‐12). Reduction in migraine days was not significant (–0.6, 95% CI –2.4 to 1.1 days; *P*=.47). App usability was impacted by software issues. No safety concerns were reported.

**Conclusions:**

Cerebri demonstrates potential in self-managed migraine treatment, with strong initial engagement and high safety. Usability issues, including technical bugs, were identified as the most important modifiable cause for the decline in adherence. This highlights the need for further app refinement to sustain user engagement.

## Introduction

Migraine is a complex and multifactorial disease and includes autonomic dysregulation and alterations in stress-related behavior [1-3]. Notably, patients with both chronic and episodic migraine have reduced heart rate variability (HRV) in comparison to healthy controls [4,5]. Biofeedback uses technology to monitor physiological processes that are typically modulated unconsciously [6-8]. Humans can learn to modulate these physiological processes by receiving continuous feedback from them [6-8]. Biofeedback training has been linked to a range of positive health effects, including reduced severity of migraine symptom burden [7,9]. A recent study has shown biofeedback’s ability to alter HRV in a migraine population [10]*,* and meta-analytical evidence suggests that biofeedback-based interventions are effective in the prophylaxis of migraine [11]. However, despite its efficacy, biofeedback is not widely used. An observational study from a specialized headache care center found that patients with migraine referred for biofeedback therapy face various obstacles to pursuing treatment, including issues related to time constraints, financial obstacles (including, but not limited to, health care insurance), and limited provider availability [12]. Traditional biofeedback training requires a trained therapist and costly and cumbersome equipment, thus limiting use to specialized clinics. Digital therapeutics and mobile health (mHealth) provide great potential for increased access to self-administered nonpharmacological treatments [13,14]. Despite this potential, we are not aware of any market-available, therapist-independent migraine biofeedback treatment with proven efficacy [15].

To fill this gap, we have developed a multimodal biofeedback system for independent home-based use. Two development and usability studies on progenitor versions of the system have already been published, but with short test periods (4 and 2 wk home testing) [16,17]. A small sham-controlled trial in adolescents tested a previous version of the medical device for 8 weeks [18]. The trial was prematurely halted due to the COVID-19 pandemic and included only 16 participants (4 sham). Poor adherence (40% in wk 5‐8) hampered the interpretation of the trial results. The purpose of this study is to assess the feasibility, usability, and safety of 12 weeks of daily use of a home-based biofeedback system as a preventative in adults with episodic migraine. The findings of the study are intended to lead to refinements in the feasibility and usability of Cerebri (version 0.1.0; Nordic Brain Tech AS) and guide study design choices for a future randomized controlled trial.

## Methods

### Study Design and Participants

The study was a single-center, open-label, one-armed interventional trial conducted at St. Olav’s University Hospital in Trondheim, Norway, from May 2022 to September 2022. The target population was adults with episodic migraine from all of Norway. The total duration of study participation for each participant was 12 weeks.

Inclusion criteria were (1) 18 years of age or older, (2) episodic migraine with or without aura diagnosed by a neurologist or physician per the International Classification of Headache Disorders, Third Edition, (3) having kept a headache diary with at least 80% adherence as part of routine clinical care in the last 28-days prior to inclusion, (4) 4 to 14 migraine attacks per 28-day period in the 3 months prior to screening, (5) at least three months of experience with smartphone and access to an iOS or Android phone at home, (6) onset of migraine before the age of 50 years, and (7) signed written informed consent.

Exclusion criteria were (1) having a continuous background headache that never disappears completely; (2) more than 14 days of headache (all types) per 28-day period; (3) participants diagnosed with trigeminal autonomic cephalalgias and neuralgias or (4) secondary headache conditions including medication overuse headache according to the International Classification of Headache Disorders 3rd edition; (5) participants with pathologies that inhibit use of the device according to the instructions for use (eg, blindness and deafness); (6) participants currently using migraine prophylaxis (pharmacological or nonpharmacological); (7) participants who have previously failed 3 prophylactic pharmacological treatments; (8) opioid use (>3 d per month) or use of barbiturates at time of screening; (9) alcohol overuse according to The International Classification of Diseases, Tenth Revision, Clinical Modification Code F10.1 or illicit drug use; (10) participation in another clinical investigation; and (11) participants unlikely to follow Clinical Investigation Plan or where treatment seems futile in the opinion of the Investigator or have demonstrated an inability to sufficiently adhere to headache diary entries (<80%).

### Interventions

**Figure 1.**
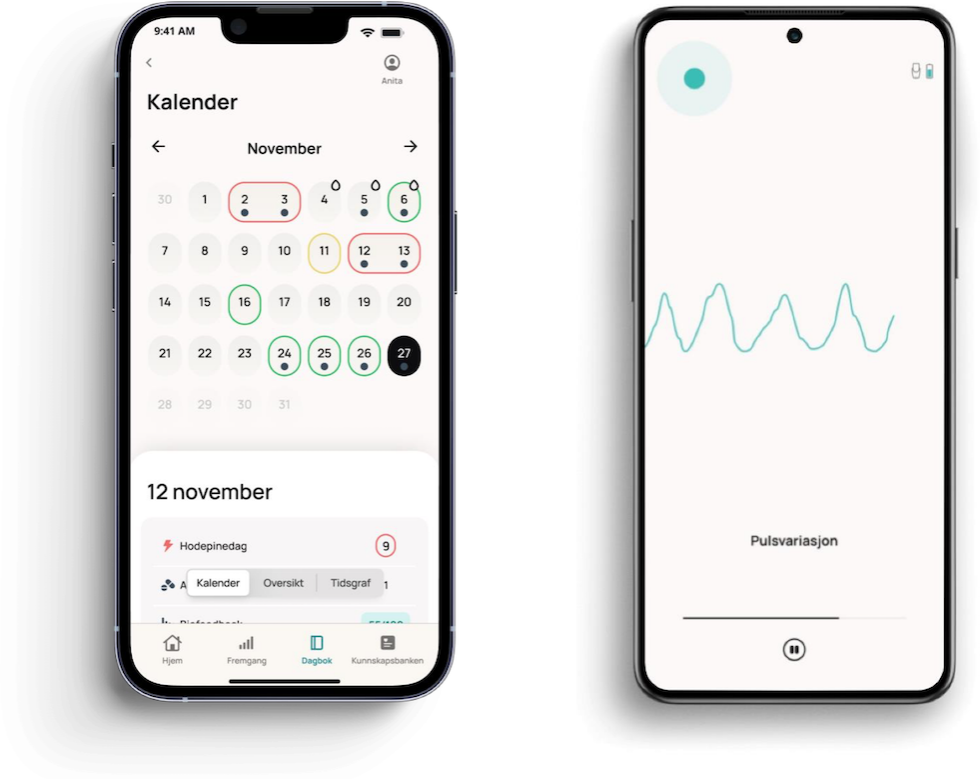
Cerebri smartphone app interface. Screenshot of eDiary and HRV biofeedback interface. eDiary: electronic headache diary; HRV: heart rate variability.

**Figure 2.**
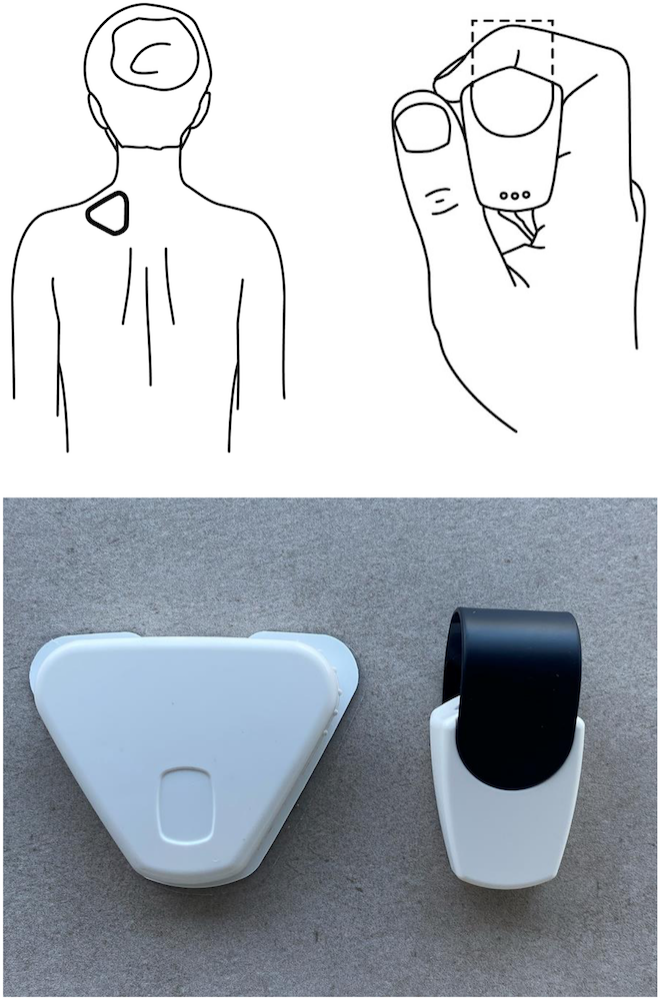
Cerebri sensors: illustrations and photograph of the finger sensor (HRV/temperature) and sEMG sensor (muscle tension). HRV: heart rate variability; sEMG: surface electromyographic.

The medical device tested in this study is a therapist-independent biofeedback system (Cerebri) consisting of a smartphone app (Figure 1) and 2 noninvasive wireless sensors (Figure 2). One sensor measures neck muscle tension as surface electromyographic (EMG) voltage from the skin over the upper trapezius muscle. The other sensor is placed on one index finger to measure HRV by photoplethysmography and peripheral skin temperature. As part of the product development, validation of sensor performance showed agreement with simultaneous control measurements made with stationary neurophysiological equipment [19]. The 12-week treatment phase with Cerebri consisted of daily biofeedback therapy sessions of a minimum of 10 minutes duration each.

Since the device is intended for independent use without the need for any supervision, no instructional sessions were offered upon receipt of the device or during the course of the study. A limited, written step-by-step on-screen guidance was provided to onboard the participant prior to the first 3 biofeedback sessions. The participants were introduced to 3 biofeedback modalities with real-time on-screen feedback: temperature biofeedback during the first session, HRV biofeedback in the second session, and EMG biofeedback in the third session. During the remaining sessions, the participants could freely alternate between the 3 modalities at their own discretion. Additional user instructions were provided in a user manual and in a library in the app, where the users could read more about biofeedback and relaxation techniques. During the biofeedback sessions, an adjustable breathing pacer was accessible to users.

### Study Follow-Up

All study visits were conducted remotely, and the sensor kit was sent by postal service. Participants accessed the app through a unique link provided via email, compatible with both Android and iOS devices. The app required an initial internet connection for download, but could subsequently operate without an active internet connection. Follow-up included 2 video consultations with a neurologist (IGW) at inclusion and the 12-week final visit; and 3 telephone follow-ups (wk 2, 4, and 8) with a study nurse. Participants were required to keep the daily eDiary integrated with the smartphone app throughout the study participation. Notifications for performing daily biofeedback sessions and diary entries were available for iPhone users, but did not work for Android users in the trial. Android users were therefore encouraged by study personnel to add a reminder manually in the integrated phone calendar to ensure this. The issue was persistent throughout the trial. Although participants were asked to register daily, the eDiary allowed registration within a 3-day interval (present day, and the 2 previous days). eDiary users were first asked if they had a headache or not. If a headache was reported, the app prompted the participant to answer additional questions about the type of headache (migraine or other), as well as the duration, intensity, and medication use. Questionnaires regarding usability and efficacy were collected via Helsenorge.

### Outcome Measures

#### Overview

The primary objective of this study was to investigate the feasibility of the Cerebri biofeedback system as a preventative treatment in adults with episodic migraine. Key secondary objectives were to (1) assess usability, (2) assess the user experience, (3) assess the safety and tolerability, and (4) evaluate data on the efficacy of the intervention, recognizing that the study was not specifically designed or powered for an efficacy analysis.

#### Assessment of Primary Outcome

The feasibility of Cerebri was evaluated by assessing the level of adherence to daily biofeedback sessions and daily eDiary entries. Adherence was defined as the percentage of completed sessions and eDiary entries per 28-day period. On a given day, adherence was defined as the completion of a 10-minute biofeedback session or a completed eDiary entry, respectively.

#### Assessment of Secondary Outcomes

Usability, satisfaction, and ease of use of the device were assessed using a Norwegian translation of the mHealth App Usability Questionnaire (MAUQ) [20]. Questionnaires were collected at weeks 1 and 12. The MAUQ contains 18 items distributed across three subscales: (1) ease of use (5 items), (2) interface and satisfaction (7 items), and (3) usefulness (6 items). Responses for each item were recorded on a 7-point scale, ranging from 1=disagree to 7=agree, with higher scores indicating better usability. Users were allowed to select the option “not applicable” for each item. Such answers were considered missing data and excluded from the analysis.

Semistructured usability interviews in a subset of participants were used to gather qualitative data about the user experience of Cerebri for product development purposes. All study participants were asked if they wished to be considered for the interview, and 5 participants declined. From a pool of 15 potential participants, 6 individuals were selected based on a set of predefined criteria (Multimedia Appendix 1). Interviews were conducted midway (at wk 6) and upon completion of the study (at wk 12).

Data on adverse events (AEs) and device deficiencies were collected at phone consultations and the final visit. All treatment-emergent AE were collected throughout study participation.

Treatment efficacy was assessed using self-reported migraine and headache days that were collected prospectively in the integrated eDiary. The number of days in each 28-day period was compared to that collected from the past 28 days prior to inclusion.

Change in self-reported, migraine-related disability was measured using the Migraine-Specific Quality of Life Questionnaire version 2.1 (MSQ) from before treatment to end-of-treatment [21]. The questionnaire contains 14 items that measure the quality of life impacts of migraine in 3 domains: “role function-restrictive,” “role function-preventive,” and “emotional function.” Participants rate their experiences on a scale of 1=none of the time to 6=all of the time. The scores for each domain in the MSQ were calculated in accordance with the license holder’s instructions, with each domain receiving a score out of 100, where a higher score indicates more favorable functioning.

The participants’ perceived change in overall health was measured by the Patient Global Impression of Change (PGI-C) at weeks 4, 8, and 12. Participants were asked to rate their current condition compared to their condition before receiving the treatment intervention on a 7-point scale, ranging from a score of 1=very much improved to a score of 7=very much worse. For descriptive purposes, participants may be classified into 4 categories: much improved (score of 1 or 2), minimally improved (score of 3), unchanged (score of 4) or disease deterioration (score of 5, 6, or 7).

### Data Analytic Strategies

Descriptive data were summarized as means with SDs or medians with IQRs according to normality distributions. Assessment of normality was based on visual inspection of histograms. Adherence rates to the eDiary and biofeedback program were calculated for each 28-day period separately. Summary statistics for MAUQ were calculated for weeks 1 and 12. Changes in the number of days with migraine and headache in each 28-day period compared to the last 28 days prior to inclusion were analyzed with a mixed logistic regression model with participant ID included as a random variable. The model estimated the odds ratio for each 28-day period. This odds ratio represented the estimated daily odds of migraine or headache in each 28-day period compared to the baseline period. The estimated number of migraines or headaches in each 28-day period and the difference from baseline were calculated based on the regression coefficients using the nlcom command in Stata (version 17.0; StataCorp LLC). The use of a mixed logistic regression model allowed the inclusion of all available daily data, also from participants who had not completed all eDiary entries during each 28-day period. The change in MSQ domain scores was analyzed using mixed linear regression models with the mean change in each domain score estimated from these models.

Semistructured interviews were held through video or phone and recorded. Transcripts were stored on the Miro (RealtimeBoard Inc) platform during analysis, where they were grouped, mixed, and sorted in several rounds to identify patterns of themes that were relevant to the usability experience.

### Ethical Considerations

The study was approved by the regional ethics committee (457654) and the Norwegian Medical Products Agency (NoMA; 22/04658‐4; Eudamed: CIV-NO-22-03-039126), and registered at ClinicalTrials.gov (NCT05454319) prior to initiation. Trial information was disseminated via multiple communication channels. Interested parties made contact by email. All participants received written information via email prior to the first study visit. Eligibility assessment and a review of the study information were performed by a consultant neurologist (IGW) with headache expertise in a video meeting. If eligible, consent was collected by digital signature via the national government-operated electronic data entry application Helsenorge (Norsk helsenett SF). Data collection for the study was initiated after electronic consent. Data were deidentified through pseudonymization. No payment was made to the study participants for their participation.

## Results

### Overview

**Figure 3.**
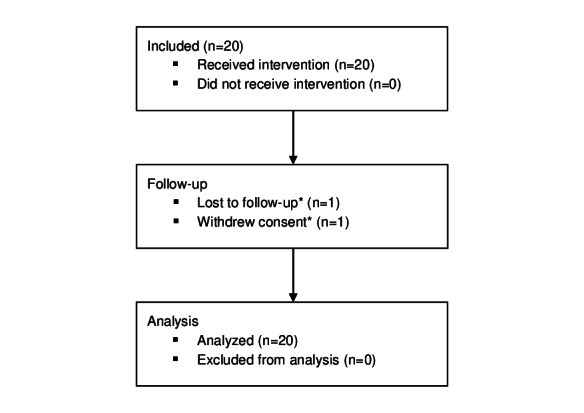
CONSORT flow diagram. Intervention: Cerebri biofeedback treatment. *Data collected from participants who withdrew or were lost to follow-up were included in the analysis. CONSORT: Consolidated Standards of Reporting Trials.

A total of 20 participants were enrolled in the study intervention. All 20 participants contributed to the evaluation of the feasibility of the Cerebri biofeedback system. One participant withdrew consent, and one was lost to follow-up (Figure 3). The majority of participants were female and in full-time employment, with a mean of 6.7 (SD 2.8) migraine days per month (Table 1). One participant was pregnant during the entire study period.

**Table 1.** Baseline characteristics.

		Value (N=20)
**Continuous variables, mean (SD)**		
Age (years)		40.4 (9.3)
BMI (kg/m^2^)		24.8 (3.1)
Baseline headache information^a^		
Number of headache days		7.7 (2.7)
Number of migraine days		6.7 (2.8)
Number of days with acute medication use		6.5 (3.1)
**Categorical variables, n (%)**		
Sex (female)		19 (95)
Pregnancy		1 (5)
Work status^b^		
Student		1 (5)
Full-time (≥100%) paid employment		15 (75)
Part-time (<100%) paid employment		1 (5)
Partial sick or medical leave		1 (5)
Sick or medical leave (100%)		1 (5)
Work assessment allowance		2 (10)
Previous use of preventive medication		4 (20)
Propranolol		1 (5)
Candesartan		1 (5)
Amitriptyline		2 (10)

a Participants all kept a headache diary for a minimum of 28 days prior to inclusion.

b One participant reported both full-time paid employment and “other governmental benefits” (parental leave). The student also received work assessment allowance and is thus registered twice.

### Primary Outcome: Feasibility

Out of 20 participants, the majority continued to regularly record information in the daily eDiary throughout the study period, with 15 (75%) participants still recording information on at least 70% of days (20 d) in weeks 9 to 12 (Table 2). The proportion of participants completing at least 70% of biofeedback sessions decreased throughout the study, with 16 (80%) participants during weeks 1 to 4, and 4 (20%) participants during the final 4 weeks (Table 2).

**Table 2.** Adherence to eDiary^a^ and biofeedback in the 3 intervals, respectively^b^.

Period		Mean (SD)	Median (IQR)	Proportion with ≥20 days recorded of all included (N=20), n (%)
**eDiary**				
Day 1‐28 (n=20)^c^		25.7 (3.8)	27 (15‐28)	18 (90)
Day 29‐56 (n=19)		25.5 (4.3)	27 (10‐28)	18 (90)
Day 57‐84 (n=18)		24.2 (4.8)	26 (12‐28)	15 (75)
**Biofeedback**				
Day 1‐28 (n=20)		20.7 (5.0)	22 (5‐27)	16 (80)
Day 29‐56 (n=19)		20.3 (5.6)	22 (6‐26)	13 (65)
Day 57‐84 (n=18)		16.7 (4.8)	17.5 (6-26)	4 (20)

a eDiary: electronic headache diary.

b The proportion of participants with adherence to intervention (eDiary and biofeedback, respectively) >20 days (=70% adherence) per 28-day period includes all participants (N=20).

c n is the number of participants in each period

### Usability

**Figure 4.**
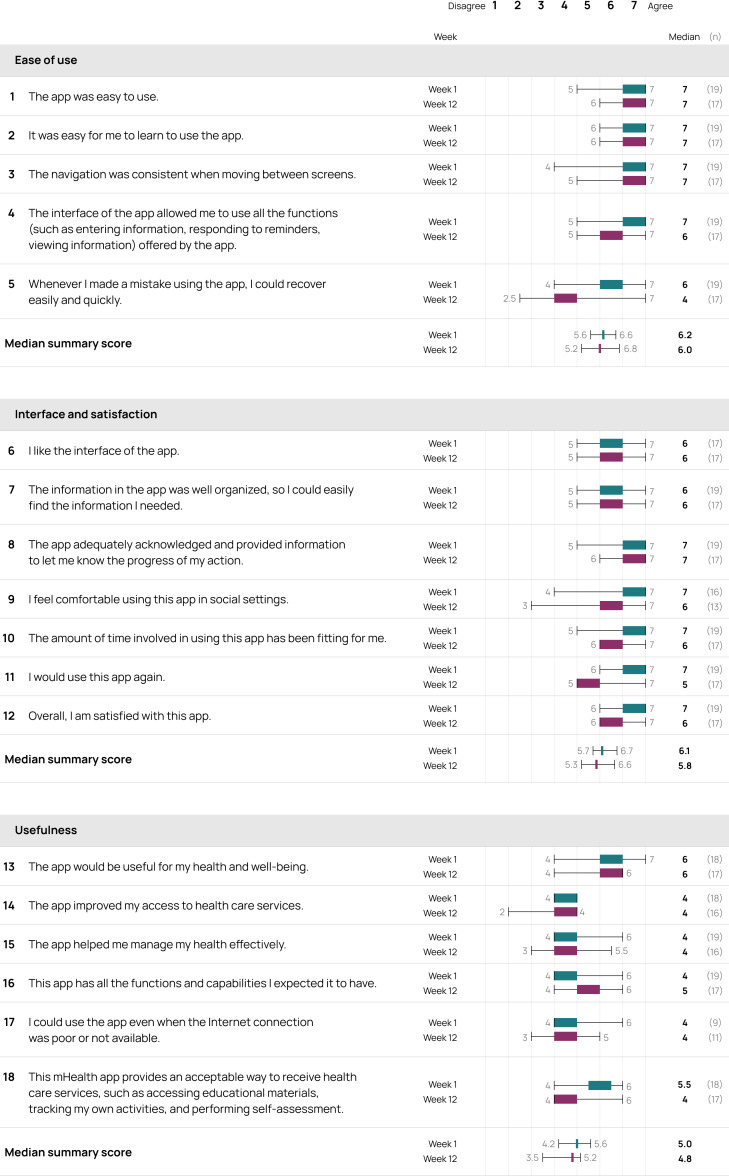
MAUQ weeks 1 (n=19) and 12 (n=17). Median scores and IQRs from each question (1-18) with median summary scores for each of the 3 domains are presented. N is the number of responses to the question, the response “not applicable” is excluded. MAUQ: mHealth App Usability Questionnaire; mHealth: mobile health.

The Cerebri app received favorable user perceptions, with MAUQ summary score for the ease of use subscale at week 1 (median score 6.2, IQR 5.6-6.6) and at week 12 (median score 6.0, IQR 5.2-6.8; Figure 4). Summary scores for the interface and satisfaction and usefulness subscales were 6.1 (IQR 5.7-6.7) and 5.0 (IQR 4.2-5.6) at week 1, and 5.8 (IQR 5.3-6.6) and 4.8 (IQR 3.5-5.2) at week 12, respectively.

The app was regarded as both easy to learn and to use (questions 1‐5), with stable median scores of 6 or 7 for almost all of these questions at both weeks 1 and 12. The exception was a notable decline in median scores from 6 (IQR 4-7) at week 1 to 4 (IQR 2.5-7) at week 12 for participants’ ability to recover after having made a mistake using the app (question 5).

MAUQ questions concerning the app’s interface and user satisfaction (questions 6‐12) also received favorable scores. However, there was a noticeable decline in the median score from 7 (IQR 6-7) at week 1 to 5 (IQR 5-7) at week 12 for question 11, which assessed users’ likelihood of using the app again. Nevertheless, overall satisfaction with the app (question 12) remained high with a median score of 7 (IQR 6-7) at week 1, and 6 (IQR 6-7) at week 12.

Furthermore, the scores for MAUQ question 13, which assessed the app’s perceived usefulness for health and well-being, remained consistently high with a median score of 6 (IQR 4-7) and 6 (IQR 4-6) at weeks 1 and 12, respectively. Meanwhile, MAUQ question 18, which evaluated whether the app provides an acceptable way to receive health care services, showed a median score of 5.5 (IQR 4-6) at week 1, decreasing slightly to 5 (IQR 4-6) by week 12.

MAUQ question 17, which addressed whether the app could be used without or with a poor internet connection, maintained a median score of 4 (IQR 4-6) at week 1 and 4 (IQR 3-5) at week 12. Notably, this item had fewer respondents compared to other questions, with 9 participants responding at week 1 and 11 participants at week 12, suggesting that not all users encountered situations where the app’s functionality under these conditions was tested.

### Usability Experience

A total of 6 participants were recruited to participate in the semistructured interviews. Five midway user interviews were completed in total. Three of these interviewees also partook in the final user interview. All interviewees reported that Cerebri was easy to both learn and use. Finding time for daily training sessions and implementing this as a daily routine activity seemed to be the most challenging part. However, despite this challenge, interviewees expressed a sense of satisfaction in being able to dedicate 10 minutes to achieving a state of complete relaxation, which they found rewarding in itself.

A concern was identified that the app focused excessively on achievements and earning high scores. Participants feared that they might not experience the intended treatment effects unless they attained the top achievements. When probed further about their reasoning, participants attributed significance to such score achievements, assuming a correlation between achieving them and experiencing a reduction in migraine symptoms.

The finger temperature parameter was reported to be preferred among users, as it was the easiest to understand whilst also providing the most concrete goal during biofeedback sessions. Output user data from all completed biofeedback sessions from all study participants identified the same preference, with a total collective screen time of 75 hours used within the finger temperature modality across a total of 132 hours in 795 sessions. In comparison, 22 and 35 hours were used in the EMG and HRV modalities, respectively. The EMG parameter appeared to provide little feedback for participants to observe and improve upon, except for one individual who reported having tense muscles and experiencing a strong connection between muscle tension and migraines. All 5 participants expressed difficulties understanding the goal of HRV measurement, how to achieve it, or both.

Several bugs in the app occurred during the second half of the study, resulting in difficulties launching the app or starting biofeedback sessions. Notably, all iPhone users were involuntarily logged out of the app at one point due to these issues. All 3 participants partaking in the final user interview mentioned bugs as a reason for reduced motivation. In total, 13 unique bugs were reported by one or more participants during the study (not limited to the interviewees). These were perceived as disruptive to the user experience and led to disruptions in use patterns, ultimately impacting user motivation and compliance. The degree to which the 3 participants experienced a drop in motivation due to technical errors was not quantified specifically during the interview, but addressed for the entire population in questions 4 and 5 in the MAQU questionnaire.

### Safety and Tolerability

Three AEs were recorded during the trial period (Multimedia Appendix 2). All AEs were assessed as nonrelated to the medical device. Thus, no adverse device effects and no serious adverse events occurred. One participant was pregnant during trial participation. No AE were registered for that participant, and the child was born healthy at term.

### Exploratory Data on Efficacy

**Figure 5.**
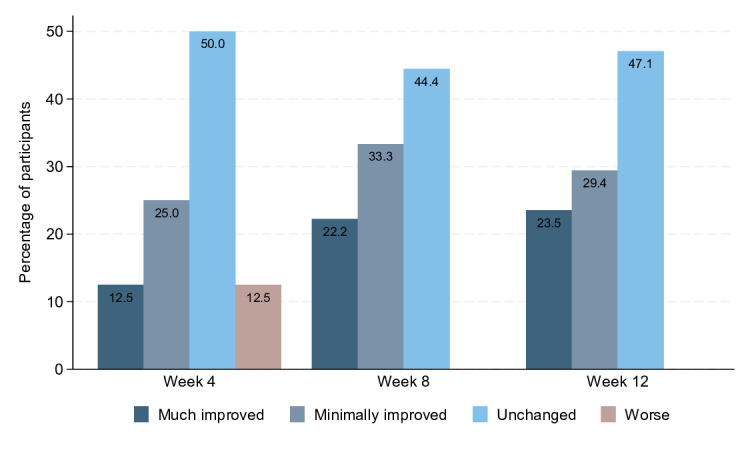
Distribution of responses to the PGI-C question at weeks 4 (n=16), 8 (n=18), and 12 (n=17). The corresponding average (SD) for weeks 4, 8, and 12 were 3.6 (0.9), 3.2 (0.8), and 3.2 (0.8), respectively. PGI-C: Patient Global Impression of Change.

We observed a statistically nonsignificant reduction in the number of migraine days compared to baseline in all three 28-day periods, but no clear change in the number of headache days (Table 3). At all timepoints, approximately 50% (8/16) of participants described overall health conditions as “unchanged” on the PGI-C questionnaire (Figure 5). However, 22% (4/18) and 24% (4/17) of participants described their condition as “much improved” in week 8 and 12, respectively. No reports of worsening conditions were reported after week 4. There was a trend toward improvements in all MSQ domains throughout the study, reaching statistical significance for the domain “role function-restrictive” in week 4 and the domain “emotional function” in week 12 (Table 4).

**Table 3.** Number of days with migraine or headache in each 28-day period and estimated change from baseline.

	Observed	Estimated from mixed logistic model			
Period	Mean^a^ (SD)	OR^b^^,^^c^	Estimated mean (95% CI)	Estimated difference from baseline (95% CI)	*P* value
**Migraine days**					
Baseline (n=20)	6.7 (2.8)	reference	6.2 (5.1 to 7.4)	reference	N/A^d^
Week 1‐4 (n=20)	5.7 (4.5)	0.80	5.2 (3.4 to 7.0)	–1.0 (–2.3 to 0.3)	.13
Week 5‐8 (n=19)	5.3 (4.0)	0.80	5.2 (3.4 to 7.1)	–1.0 (–3.0 to 0.9)	.31
Week 9‐12 (n=18)	5.9 (4.2)	0.87	5.6 (3.8 to 7.4)	–0.6 (–2.4 to 1.1)	.47
**Headache days**					
Baseline (n=20)	7.7 (2.7)	reference	7.5 (6.3 to 8.7)	reference	N/A
Week 1‐4 (n=20)	8.1 (4.2)	1.07	7.9 (6.1 to 9.6)	0.4 (–0.9 to 1.6)	.58
Week 5‐8 (n=19)	8.0 (4.1)	1.04	7.7 (5.9 to 9.6)	0.2 (–1.7 to 2.2)	.81
Week 9‐12 (n=18)	7.4 (4.1)	0.97	7.3 (5.5 to 9.1)	0.2 (–2.1 to 1.7)	.85

a Mean number of days based on available information in each 28-day period. For participants who did not complete the headache diary on all 28 days, the number of migraine or headache days was divided by the number of days with complete headache diary entries multiplied by 28 to obtain the number of days the participant would have been expected to have a migraine or headache.

b OR: odds ratio.

c A mixed logistic regression model was used to estimate the OR for each 28-day period. This OR represented the estimate OR compared to the daily odds of migraine and headache for each 28-days period. The estimated 28-day migraine and headache differences was calculated based on the regression coefficients and using the nlcom command in Stata.

d Not applicable.

**Table 4.** Migraine-specific quality of life questionnaire scores at different timepoints and differences from baseline: presented according to the 3 domains.

	Observed		Estimated difference from baseline	
Week	Mean (SD)	Median (IQR)	Mean difference (95% CI)	*P* value^a^
**Role function—restrictive domain**				
Baseline (n=19)	61.2 (14.2)	60.0 (51.4-74.3)	reference	N/A^b^
Week 4 (n=16)	68.0 (10.2)	68.6 (60.0-77.1)	6.8 (0.6 to 13)	.03^a^
Week 8 (n=18)	69.8 (15.3)	70.0 (60.0-77.1)	8.6 (–0.2 to 17.4)	.06
Week 12 (n=17)	67.7 (17.9)	62.9 (60.0-80.0)	6.5 (–4.4 to 17.4)	.24
**Role function—preventive domain**				
Baseline (n=19)	75.8 (17.2)	75 (60-95.0)	reference	N/A
Week 4 (n=16)	81.6 (13.3)	85 (77.5-90.0)	5.7 (–1.8 to 13.2)	.14
Week 8 (n=18)	85.0 (15.9)	95 (65.0-95.0)	8.9 (–0.9 to 18.7)	.07
Week 12 (n=17)	80.6 (14.6)	80 (75.0-95.0)	4.6 (–5.8 to 15)	.39
**Emotional function domain**				
Baseline (n=19)	66.0 (20.2)	73.3 (53.3-80.0)	reference	
Week 4 (n=16)	72.9 (18)	76.7 (63.3-86.7)	5.7 (–1 to 12.5)	.10
Week 8 (n=18)	74.4 (18)	76.7 (60.0-86.7)	7.6 (–1.7 to 16.9)	.11
Week 12 (n=17)	76.9 (18.3)	80.0 (66.7-93.3)	9.9 (0.3 to 19.5)	.04^a^

a Indicate statistical significance (*P*<.05).

b Not applicable.

## Discussion

### Overview

This study was designed to address the feasibility of the home-based, therapist-independent biofeedback system Cerebri as a preventive treatment in adults with episodic migraine to serve as a preparatory step toward a clinical efficacy trial. Additionally, major emphasis was given to assessing the usability and the safety of the invention.

### Principal Findings

Daily biofeedback session adherence declined throughout the course of the study from 80% in weeks 1‐4 to 20% in weeks 9‐12. The decline in diary adherence was much less pronounced, with a reduction from 90% to 75% in the same time interval. Low adherence is a common concern for nonpharmacological headache treatments [22]. This is also true for biofeedback treatment, as seen in a previous pilot on a precursor to the Cerebri system [18], as well as in another mHealth app for biofeedback intervention in migraine [23]. In this study, several causes for low adherence were identified.

First, software bugs in the last part of the study period hampered adherence, causing at one point all iPhone users to be logged off the app. Second, as eDiary adherence was better, software malfunction cannot be the sole cause for the declining biofeedback adherence. Usability assessments, including MAUQ scores, identified several other software elements that should be improved. Details were elucidated in semistructured interviews held in a subset of participants for product development purposes. The achievement features conveyed unrealistic goals for some participants, and failure to reach them was misinterpreted as a failed session by some. In addition, the goal of the HRV biofeedback training was difficult to understand.

This study was not designed to assess efficacy, and outcomes must be interpreted with caution. There was an observed tendency for improvements in several headache parameters, including quality of life measures. Notably, a not statistically significant average reduction in migraine days was observed across all three 28-day intervals compared to baseline, with an estimated reduction of 1 migraine day in weeks 1‐4 and weeks 5‐8. On PGI-C scoring, 24% (4/17) of participants described their overall health condition as “much improved” at week 12.

The intervention was safe, with no device-related or serious AE.

### Interpretation

This study highlights the potential for therapist-independent, multimodal biofeedback systems to address unmet needs in migraine management, despite challenges in adherence. Adherence challenges, observed in this and prior studies [18,23], emphasize the need for robust technology platforms and user-centered design to secure long-term engagement, particularly in home-based interventions.

A possible explanation for the decreasing adherence to the biofeedback sessions, despite consistently high eDiary adherence, is that users experienced some sort of improvement from the therapy, and therefore, adjusted the frequency of treatment sessions while retaining the treatment effect. However, it may also be an expression of a lack of perceived effect, and either way, user interviews did not identify this as a major mechanism.

There is no consensus on the optimal treatment duration or frequency of home-based mHealth behavioral interventions in migraine. Traditional therapist-dependent biofeedback is typically provided as hour-long sessions once or twice weekly, sometimes with intermittent home-training of shorter duration. In a recent pilot study examining a home-based single-modality surface EMG biofeedback intervention for episodic migraine, participants engaged in weekly sessions lasting 40‐45 minutes under the remote guidance of a biofeedback therapist over a 6-week period [24]. Participants were instructed to do additional sessions of 30 minutes 3 times a week and keep track of their home practice. Participants attended on average 4.8 (SD 1.2) of the 6 sessions, whereas adherence to the therapist-independent sessions was not reported in the paper.

It can be hypothesized that increased treatment duration yields larger treatment effects. For home-based therapist-independent treatment, long sessions may hamper adherence, whereas short sessions may reduce potential efficacy. In a pilot study on a combined HRV-biofeedback-virtual reality device, participants attended an initial in-person session, after which they were instructed to perform sessions at home a minimum of 3 times per week, with each lasting at least 10 minutes [10]. There was an attrition rate of 9 out of 25 participants in the intervention group, compared to 3 out of 25 in the control group. No statistically significant reduction in headache days between the groups was observed. Results from this study suggest the possibility of significant dropout even with less frequent sessions. Therefore, increased session duration or frequency may not necessarily be determining factors for adherence to the intervention. This pilot study did identify adherence challenges, though other underlying mechanisms seem more important than session duration and frequency itself. Future work should empirically investigate optimal treatment duration and frequency.

Electronic behavioral interventions are dependent on sufficient user satisfaction to secure adherence. The overall positive perceptions of the app’s ease of use and utility underscore Cerebri’s potential to serve as an accessible, self-administered treatment option. However, the decline in median scores for certain usability aspects, particularly users’ likelihood of using the app again, could signal the need to revise and improve certain elements of the app’s design. On the other hand, a lack of perceived clinical effect could also explain such a finding. Regardless, addressing concerns related to usability features is crucial to maintaining patient compliance. To that end, revisions related to app achievement features should be a priority to mitigate misunderstandings that may affect participants’ motivation and perceived treatment effectiveness. Other strategies to reinforce habit formation should be explored.

Furthermore, our results suggest that the app interface of the HRV biofeedback modality should be revised to facilitate the correct performance of training within this modality. This could be achieved by including a visual guiding line matching the optimal sinus curve for the selected breathing frequency. In addition, improving HRV-related textual information in the app may increase user accessibility.

### Limitations

This pilot study has several limitations. First, the sample size of 20 participants makes the study underpowered to make claims regarding efficacy. Additionally, the absence of a control group limits our ability to attribute effects specifically to the intervention. A notable limitation of our qualitative analysis is the small sample size in the final user interviews, where only 3 participants participated. Additionally, the sequential selection of interview participants during the week 4 follow-up appointments could have introduced a selection bias as they were not randomly drawn. However, we aimed to include a diverse representation of age, gender, and adherence, and thus, opted for a sequential selection.

Participants were biofeedback naïve, but had already kept a headache diary with a minimum of 80% adherence in the 4 weeks prior to inclusion as part of routine clinical care, and were experienced smartphone users. Thus, study results may not be representative of a smartphone and eDiary naïve population.

Furthermore, as the study included only individuals with episodic migraine, caution is advised when inferring results to other patient groups, including chronic migraine or other primary headache disorders.

### Conclusions

Cerebri is a unique multimodal, app-based, and therapist-independent biofeedback intervention that, in this study, underwent a rigorous feasibility and usability evaluation. The main feasibility obstacle was declining adherence to daily biofeedback sessions throughout a 12-week treatment period. On the other hand, eDiary adherence was consistently high. Usability issues and software bugs were identified and will be addressed in the final product development of Cerebri prior to a pivotal randomized controlled trial.

## Supplementary material

10.2196/59622 Multimedia Appendix 1 Predefined criteria for semistructured usability interviews.

10.2196/59622 Multimedia Appendix 2 Registered adverse event (AE).

## Abbreviations

AE: adverse event
EMG: electromyographic
HRV: heart rate variability
MAUQ: mHealth App Usability Questionnaire
mHealth: mobile health
MSQ: Migraine-Specific Quality of Life Questionnaire
PGI-C: Patient Global Impression of Change
