# Supplementary material for: Feasibility of a 12-Week, Therapist-Independent, Smartphone-Based Biofeedback Treatment for Episodic Migraine in Adults: Single-Center, Open-Label, 1-Armed Trial
Source: JMIR Hum Factors. 2025 Jun 9;12:e59622. doi: 10.2196/59622 (PMC12169497; doi:10.2196/59622)
Supplement: Multimedia Appendix 1 [file humanfactors-v12-e59622-s001.docx]

During the follow-up appointment in week 4, the nurse will select 6 individuals from the patient list, starting from the top of the list, and identifying patients who meet the following criteria. The primary goal is to involve patients in the interview; it is not essential to strictly adhere to the criteria.

1. The patient must be willing to participate in a video call.
2. The patient must be willing to be recorded on audio.
3. The following criteria may overlap:
4. At least one male.
5. At least one female.
6. At least one in the age group «30-45»
7. At least one in the age group «over 45»
8. At least one with low adherence to the treatment program.
9. At least one with high adherence to the treatment program
