# Supplementary material for: Feasibility of a 12-Week, Therapist-Independent, Smartphone-Based Biofeedback Treatment for Episodic Migraine in Adults: Single-Center, Open-Label, 1-Armed Trial
Source: JMIR Hum Factors. 2025 Jun 9;12:e59622. doi: 10.2196/59622 (PMC12169497; doi:10.2196/59622)
Supplement: Multimedia Appendix 2 [file humanfactors-v12-e59622-s002.docx]

One participant experienced localized numbness in three fingers after a work-related incident, whilst another participant (without prior known allergies) suffered what was believed to be an allergic reaction to a cosmetic facial product, with symptoms including facial swelling, erythema and itching. One participant had covid-19 during the study period.

### 
